# 3-T MRI implant safety: heat induction with new dual-channel radiofrequency transmission technology

**DOI:** 10.1186/s41747-018-0040-y

**Published:** 2018-04-17

**Authors:** Nadja A. Farshad-Amacker, Daniel Nanz, Arjun Thanbanbalasingam, Gustav Andreisek, Mathias Nittka, Roger Luechinger

**Affiliations:** 1 Institute of Diagnostic and Interventional Radiology, University Hospital of Zurich, Raemistrasse 100, 8091 Zurich, Switzerland; 2 Siemens Healthcare GmbH, Erlangen, Germany; 3 Institute for Biomedical Engineering, University and ETH Zurich, Zurich, Switzerland

**Keywords:** Magnetic resonance imaging (MRI), Circularly polarized transmission, Elliptically polarized transmission, Radiofrequency heating, Metallic implant

## Abstract

We aimed to investigate whether different transmission settings of the dual-transmit technology may influence the amount of heat induction around an implant material dependent on its location within the magnetic field. Metallic hip implants were positioned in the magnet of a 3-T scanner at various lateral offset positions in relation to the magnetic axis in a body-phantom tank filled with polyacrylic acid gel. The temperature increase close to the implants was measured during turbo spin-echo scanning using dual-channel parallel radiofrequency (RF) transmission with circular in comparison to elliptic RF polarization. Circularly polarized transmission (CPT) induced higher temperature increases (maximum 6.2 °C) than elliptically polarized transmission (EPT) (maximum 1.5 °C). The heat induction was dependent on the distance to the isocenter with increased heating by increased distance to the isocenter. EPT showed lower heating around implants compared to the CPT as commonly used in single-transmission system; further, less heating was observed for both transmission settings closer to the magnet isocenter.

## Key points

- Elliptically polarized transmission showed lower heating around implants compared to the circularly polarized transmission
- Heating of metallic implants was dependent on the distance to the isocenter
- Less heating was observed closer to the isocenter of the MR scanner for both transmission settings

## Background

Recent implementations of metal-artifact reduction sequences in magnetic resonance imaging (MRI) have allowed the evaluation of anatomical structures close to metallic implants [1–3]. These sequences steadily become more important in daily clinical use given the strongly increasing rates of worldwide prosthesis implantations and of health issues in an aging population. The Food and Drug Administration now recommends to include metal-artifact reduction sequences in the preoperative evaluation of symptomatic patients with metal-on-metal hip implants [4].

However, there are safety issues associated with the examination of patients with metallic implants [5]. The principles of heat induction by MRI radiofrequency (RF) transmission are well known [6, 7]. The trend towards MRI scanning at high field (≥ 3 T), beyond challenges of optimizing image contrast and minimizing artifacts, also accentuates the problem of guaranteeing patient safety [8]. When comparing nominally equal image-acquisition pulse sequences, the RF energy irradiated at 3 T can be up to four times higher than at 1.5 T, while the limits for the estimated absorbed energy are the same at both field strengths [9]. The shorter RF wavelength at 3 T can cause spatially inhomogeneous image contrast. Thus, attempts were made to optimize the RF transmitting field by using a multi-transmit system, such as the TimTX TrueForm technology (Siemens Healthcare, Erlangen, Germany). It allows dual-channel parallel transmission via a two-port birdcage body coil and promises a more uniform RF field throughout the imaged body volume, with improved B_1_ homogeneity (RF shimming) and, thus, also the potential to minimize standing-wave artifacts [10–13]. In part, the improvements are reached by (optionally) switching from the conventional circular field polarization to an elliptically polarized B_1_ field, whereas single-channel RF transmission systems only allow circular RF polarization [14]. When scanning the thorax or abdomen, with their elliptic trans-sectional geometries, the technology potentially improves image quality by channel-specific, independent adaptation of the two RF channel amplitudes according to pre-established optimized patterns (elliptic TrueForm). Such adaptations have been shown to have consequences for RF-induced temperature increases [15, 16].

While manufacturers, must demonstrate that their systems reliably stay within the limits specified in the International Electrotechnical Commission 60601-2-33 standard [17], potential additional complications that may occur in the presence of metallic implants are not well investigated or documented. The MRI safety labels are defined by the American Society for Testing and Materials (ASTM) F2503-13 [18], which helps the implant manufacturer in guiding them which test for which implant is needed in respect to test for safety.

Radiofrequency heating tests are currently only done using circular polarization. To obtain an MRI-conditional label, implants need to be tested for RF heating at all field strengths, since the effects may vary, e.g., due to varying resonance effects at different resonance frequency. Ideally, the safety should be established for all RF transmission schemes at each field strength.

Therefore, the purpose of this phantom study was to comparatively assess RF heating effects at 3 T using the circularly polarized RF transmission (CPT) in comparison to the elliptically polarized RF transmission (EPT) setting in the presence of metallic hip implants.

## Methods

### Phantom settings and preparation

**Fig. 1:**
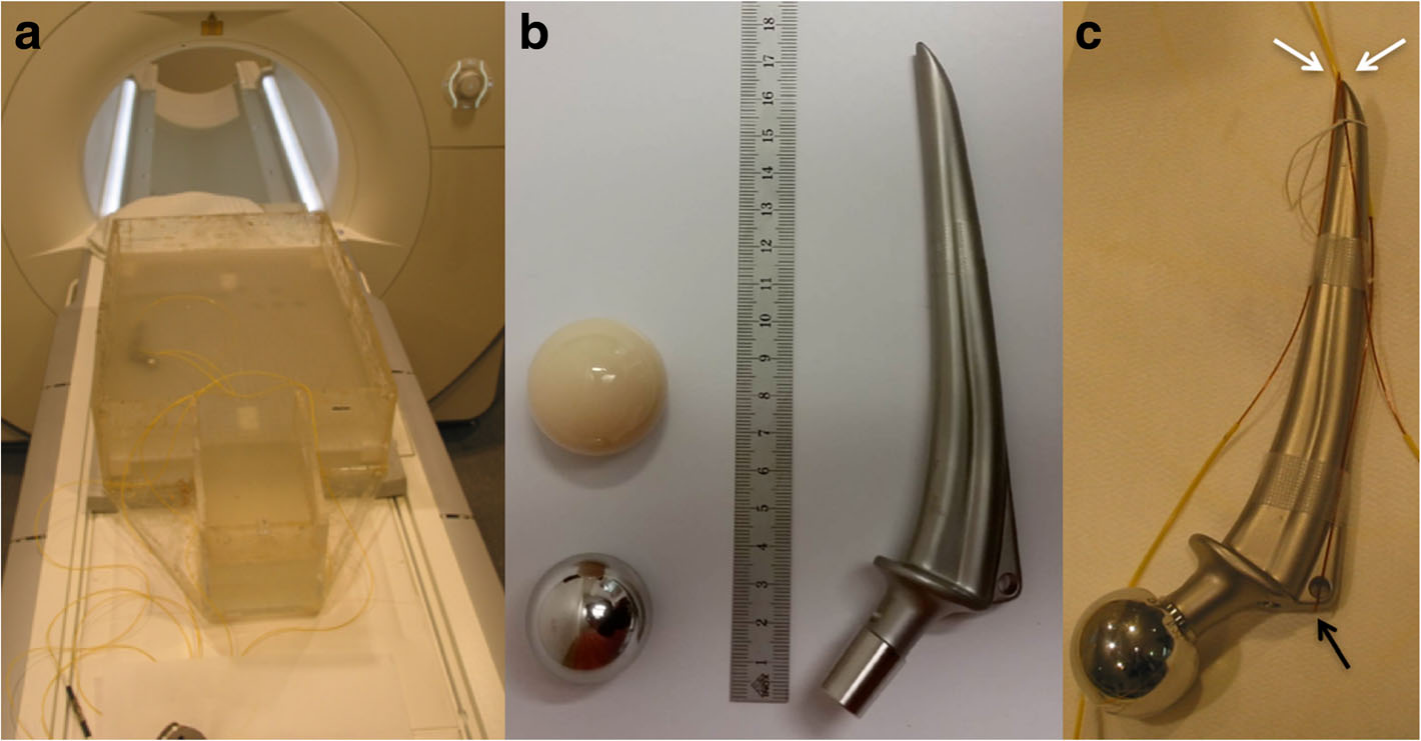
Images of the implants and phantom. **a** Phantom tank filled with polyacrylic acid gel including a hip prosthesis placed on the grid. **b** Stainless steel and ceramic head as well as cobalt-chrome (CoCr) stem with dimensions. **c** CoCr stem with the stainless steel head and the attached temperature probes. Two temperature probes were positioned at the lower end of the stem (white arrows) and one at the upper end of the stem (black arrow)

MRI-induced heating was measured in accordance with guideline of the ASTM F2182-11a [18], using a body-phantom tank (45.5 cm × 65.5 cm) filled with an 8-cm-deep layer of an polyacrylic acid gel, which showed a conductivity at low frequency and 21 °C of 0.47 ± 0.04 S/m (mean ± standard deviation) (Fig. 1a).

A plastic grid was placed 4 cm above the bottom of the tank, in the middle of the gel layer (Fig. 1a). A hip implant with a stem made of cobalt-chrome (CoCr) (Fig. 1b) and one of two heads made of stainless steel or ceramic (Fig. 1b) were positioned on the grid (Fig. 1a). Further, a titanium cup was available. The implant materials were supplied by Brehm (Weisendorf, Germany). The implants were aligned as much as possible along the magnet B_0_ direction on the plastic grid (Fig. 1a). Temperatures during MRI scanning were measured with a four-channel fiber-optic conditioner with four sensors and a polyamide 0.6-mm tip (Reflex, Neoptix, Quebec, Canada). Two temperature probes were attached at the lower end of the stem, one at the upper end of the stem (Fig. 1c) and one at the periphery of the tank on the contralateral side within the gel to provide a reference baseline temperature.

### MRI protocol

All implants were scanned with a T2-weighted turbo spin-echo sequence on a 3-T MRI scanner (Magnetom Skyra, Siemens Healthcare, Erlangen, Germany). The technical parameters were the same for EPT and CPT setting and are summarized in Table 1. For scanning, a 40-year-old patient weighting 75 kg was entered in the scanner interface. In the elliptic RF excitation setting, the amplitude voltage of one transmit channel was automatically reduced by the scanner by approximately 54% in relation to that of the second channel.

**Table 1:** Scan parameters

	TSE circularly polarized	TSE elliptically polarized
TR (ms)	1600	1600
TE (ms)	49	49
Turbo factor	40	40
NAV	12	12
FOV (cm^2^)	50 × 40.6	50 × 40.6
Matrix	512 × 416	512 × 416
Flip angle	150	150
RF transmission polarization setting	circularly	Elliptically
wbSAR (W/kg)	1.042	0.75

*TSE* turbo spin-echo, *TR* repetition time, *TE* echo time, *NAV* number of averages, *FOV* field of view, *RF* radiofrequency, *wbSAR* whole-body averaged specific absorption rate

### Temperature measurements

As a pre-test reference, the empty tank, without any implant, was scanned using the CPT setting and the EPT setting for 2 min each. The two temperature probes were placed on the grid at the two sides of the tank, each with a 20-cm lateral offset from the isocenter of the scanner.

RF heating effects around implants are known to potentially strongly differ at implant positions that are axially symmetric to the left and right of the magnetic axis through the magnet’s isocenter. In the current study, after initial “very-bad-case” measurements conducted with the implant symmetrically positioned on both sides of the magnet bore, subsequent experiments were restricted to implant positions on the side that produced the higher heating. For the initial measurements, the CoCr stem with the attached stainless steel head was positioned at a 20-cm lateral offset from the isocenter of the magnetic axis on both sides in successive experiments that both were repeated with CPT and EPT, respectively.

Subsequently, the temperature increase near the CoCr stem with the mounted ceramic head was measured as a function of the left lateral offset. The implant was positioned at lateral offsets from the isocenter of 20, 16, 12, 8, and 4 cm; at each offset the induced temperature increase was measured for both CPT and EPT transmission settings.

In addition, the temperature increase induced by the CoCr stem with mounted stainless steel head and titanium cup was measured and compared with that induced by the CoCr stem/ceramic head combination at a 20-cm left-lateral offset from the isocenter. All measurements were repeated with both CPT and EPT transmission settings.

### Data recording and presentation

Data were recorded using Excel (Microsoft, Redmond, WA, USA) and presented descriptively. Illustrations were created using software PRISM (Version 7, GraphPad software, La Jolla, CA, USA).

## Results

In the 2-min baseline experiment without any implant in the phantom, a maximal temperature increase of 0.5 °C was measured with CPT, with a whole-body averaged specific absorption rate (wbSAR) of 1.042 W/kg, while with the EPT (wbSAR 0.750 W/kg) no relevant temperature increase was observed. The temperature increase on the left side (view from the patient table to the scanner) was higher compared to the right side (4.3 °C versus 1 °C; wbSAR 1.042 W/kg) using CPT.

**Fig. 2:**
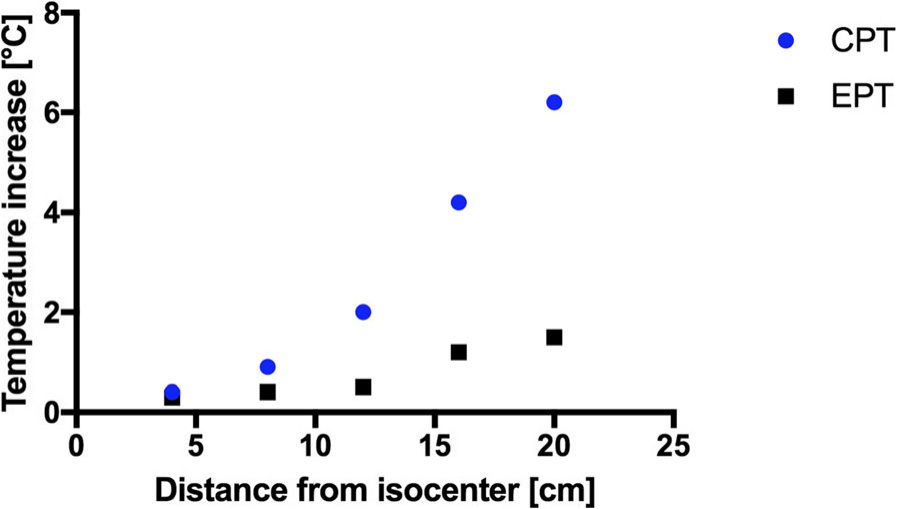
Temperature increase of one of the implant tips attached at the lower end of the cobalt-chrome (CoCr stem with the ceramic head as a function of the lateral offset of the implant from the isocenter. Note the steadily increasing heating for increasing lateral offsets. The effects were clearly larger for radiofrequency (RF) transmission with circularly polarized (CPT) than with elliptically polarized transmission (EPT)

In general, more severe heating was measured using the CPT than using the EPT setting (Table 2, Fig. 2), with the highest temperature increase measured at the greatest distance from the isocenter (6.2 °C versus 1.5 °C) (Table 2, Fig. 2). CoCr stem together with the stainless steel head and the titanium cup compared to the CoCr stem together with the ceramic head and the titanium cup showed slightly more severe heat induction when using the CPT setting (5.7 °C versus 5.4 °C; wbSAR 1.042 W/kg), while it showed slightly less heat induction when using the EPT setting (1.2 °C versus 1.5 °C; wbSAR 0.750 W/kg) at a 20-cm lateral offset from the isocenter on the left side.

**Table 2:** Induced temperature increase, *ΔT*, of the cobalt-chrome (CoCr stem with the ceramic head at different lateral offsets and under circular versus the elliptic transmission

Lateral offset from magnetic axis (cm)	RF transmission polarization mode	B_1_ + rms (μT)	*ΔT* (°C)	wbSAR (W/kg)
20	Circular	3.248	6.2	1.042
	Elliptic	3.335	1.5	0.75
16	Circular	3.248	4.2	1.042
	Elliptic	3.335	1.2	0.75
12	Circular	3.248	2	1.042
	Elliptic	3.335	0.5	0.75
8	Circular	3.248	0.9	1.042
	Elliptic	3.335	0.4	0.75
4	Circular	3.248	0.4	1.042
	Elliptic	3.335	0.3	0.75

*RF* radiofrequency; *B*_*1*_ *+ rms* root-mean-square value of the magnetic resonance imaging (MRI) effective component of the RF magnetic (B_1_) field, *μT* microtesla, *ΔT* induced temperature increase; *°C* degree Celsius; *wbSAR* whole-body averaged specific absorption rate, *W/kg* Watts per kilogram

## Discussion

In this study, we investigated whether different dual-channel transmission settings, such as the CPT versus the EPT, may determine different heat induction around an implant material dependent on its location within the magnetic field. While the heat induction steadily increased with the lateral offset from the isocenter for both settings, it was consistently higher for CPT than for EPT. To the best knowledge of the authors, this is the first study that reports a reduced heat induction through the use of an EPT in comparison to conventional CPT. It is also the first report to highlight that EPT reduces the heat induction near implants placed with a lateral offset from isocenter in comparison with CPT.

The main driving force for the development of dual-channel, parallel RF transmission technology was the reduction of standing-wave artifacts in MRI of the abdomen and thorax [12, 13]. However, the technique was also reported to reduce artifacts and improve image quality in other body regions, which was mainly explained with a better B_1_-field homogeneity [10] or a decrease in locally induced RF currents resulting in decreased shading artifacts around metallic implants [19].

For MRI-conditional devices, the safety is tested for traditional, single-channel, RF body coils. In our measurements, conducted in a phantom tank and with implant orientations as recommended for RF heating tests according to the ASTM standard F2182-11a [18], the EPT resulted in consistently less heating than the CPT. However, our findings cannot be generalized to the general assumption that EPT would always be preferable to, and safer than, CPT. Actual electric field patterns in a patient’s body depend on many factors that are hard to control in clinical examination. Certain configurations may lead to higher heating with EPT than with CPT. For example, Murbach et al. demonstrated that, in the absence of implants, EPT may generate a higher temperature increase near the fetus in pregnant women than CPT [16].

However, the presented results are important not only for clinical daily use, where the EPT setting should be preferably used if scanning implants, but also for safety test measurements to avoid false underestimations of heat induction around metallic objects when are conducted with EPT instead of CPT. If in vitro RF heating tests are performed following the ASTM standard F2182-11a [18]. However, using EPT instead of CPT (as required), the results could strongly underestimate the potential RF heating. Special care is needed, since some dual-transmit MR system from other vendors might force the use of EPT setting, which cannot be overruled in clinical software.

Some study limitations need recognition. First, all the temperature measurements of implant-related heating were performed over a time period of 2 min. This is a shorter time than the duration of many clinical acquisition sequences. However, our goal was not to provide evidence for the certification of an MRI-conditional label for the used implants, but to relatively compare the effects of two RF transmission settings. The scan duration (2 min) was chosen because an assessment of the relative heat induction was considered sufficient for a comparison of the two RF transmission settings. The temperature increases reached after 2 min were large enough for a robust quantification of the effects but small enough to keep the required cool-down periods between two successive experiments sufficiently short to allow the realization of this study. A scan duration of 15 min would result in a temperature increase of about twice as high, but deposit 7.5 times more energy with thus much longer waiting time. This would only lead to a higher likelihood of alteration of the implant or the temperature sensors and, therefore, potentially generate larger errors. In addition, we only used the body coil for RF transmission. The use of transmit head or knee coils may produce different results, while the choice of the receive coil seems of minimum relevance in this context. Finally, this was only a phantom study. Studying such effects in living humans is very challenging, and the positioning of temperature probes next to the implant is not realistic.

In conclusion, in this phantom study, comparatively testing different dual-channel transmission settings (CPT versus EPT), we demonstrated that the heat induction around metallic implants increases with increasing lateral implant distance from the isocenter and is more severe using RF with CPT than with EPT. However, further studies, including simulations and evaluations on humans, are needed to verify and generalize our findings.

## Abbreviations

ASTM: American Society for Testing and Materials
CoCr: Cobalt-chrome
CPT: Circularly polarized transmission
EPT: Elliptically polarized transmission
MRI: Magnetic resonance imaging
RF: Radiofrequency
wbSAR: Whole-body averaged specific absorption rate
